# High-power modelocked thin-disk oscillators as potential technology for high-rate material processing

**DOI:** 10.1515/aot-2021-0045

**Published:** 2021-11-25

**Authors:** Yicheng Wang, Sergei Tomilov, Clara J. Saraceno

**Affiliations:** Photonics and Ultrafast Laser Science, Ruhr Universität Bochum, Universitätsstrasse 150, 44801 Bochum, Germany

**Keywords:** fast material processing, high-power laser, ultrafast laser

## Abstract

High average power femtosecond lasers have made spectacular progress in the last decades – moving from laboratory-based systems with maximum average powers of tens of watts to kilowatt-class mature industrial systems in a short time. The availability of such systems opens new possibilities in many fields; one of the most prominent ones that have driven many of these technological advances is precise high-speed material processing, where ultrashort pulses have long been recognized to provide highest precision processing of virtually any material, and high average power extends these capabilities to highest processing rates. Here, we focus our attention on one high-average power technology with large unexplored potential for this specific application: directly modelocked multi-MHz repetition frequency high-power thin-disk oscillators. We review their latest state-of-the-art and discuss future directions and challenges, specifically with this application field in mind.

## 1 Thin-disk laser technology

### 1.1 Introduction

**Figure 1:**
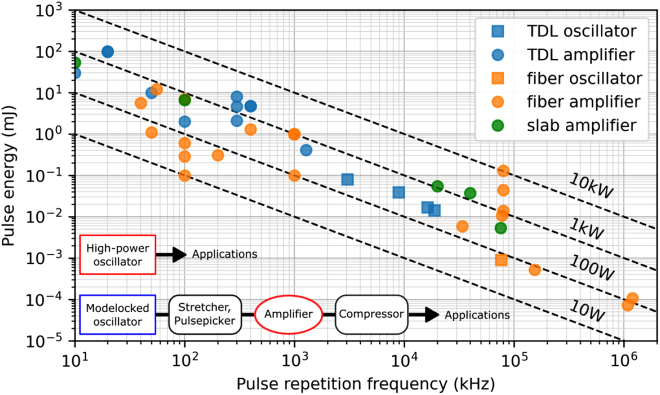
State-of-the-art high-power ultrafast lasers at high pulse repetition frequency >10 kHz illustrating latest advances obtained with Yb-doped ultrafast laser systems in the fiber, slab and disk geometry including amplifiers and oscillators. For each technology the performance of high-power oscillators is highlighted.

Ultrafast lasers have been at the forefront of many breakthroughs in fields as diverse as physics, chemistry and biology as well as mechanical and electrical engineering – including several Nobel Prize winning works – since their invention in the 1990s [1]. They have progressed from specialized laboratory tools to commercial equipment extensively used in both settings. One frontier that has evolved particularly fast in the last decade is their average power – evolving from typical values in the few watts to multi-kilowatt systems in a short time. This fast-paced progress was enabled by laser technologies based on Yb-doped gain materials: Yb- as an active ion in most commonly used garnet hosts such as YAG and LuAG has inherent advantages for average power scaling – a typically long upper-state lifetime combined with large emission cross-sections, a very small quantum defect and pumping wavelengths accessible with very high-power diode pumps. These inherent spectroscopic advantages ideally complement geometries with better cooling properties than the traditionally used rods, making use of a better surface-to-volume ratio. Based on these architectures, lab-based ultrafast lasers based on fibers, slabs and thin-disks have now long surpassed the kilowatt average power level and even very recently the 10-kW milestone [2], [3], [4], [5], see Figure 1.

Commercially available systems are following this trend and nowadays multihundred-watt levels systems are becoming increasingly common in ultrafast laboratories and in industrial scenarios. The wider availability of such systems opens new possibilities in many fields; one of the most prominent ones being the one of precise high-speed material processing. In fact, the use of ps and sub-ps pulses for ablation has been well-recognized for a long time for offering highest precision and processing quality compared to more traditional ns lasers [6]. For ultrashort pulses where the pulses duration is below the electron-phonon coupling time (a few ps in case of metals [7]), the removal rate or volume ablation rate per average power typically has an optimal fluence above the threshold fluence as a result of the logarithmic ablation law [7], [8], [9], and access to high average power at a given pulse energy (and at given focusing condition corresponding to the ideal fluence) allows us to operate at correspondingly higher pulse repetition frequency according to *P*_av_ = *E*_p_·*f*_rep_. Please note that we are using the term pulse repetition frequency throughout the text to distinguish the frequency of the pulses coming from the laser source from the machining repetition rate which can be lower than the pulse repetition frequency (although in most of the literature these two terms are identical).

New ultrafast laser technologies with multihundreds of watts to kilowatts allow operating with tens to hundreds of MHz laser pulse repetition frequency, offering the possibility to operate at extremely high speeds when using fast scanning methods [10]. Since such state-of-the-art scanning systems operate with repetition rates of tens of MHz [11], we focus our attention here on latest developments of high-average power ultrafast lasers in this pulse repetition frequency class. In particular, we aim here to highlight one high-average power technology with unexplored potential for this specific application: directly modelocked multi MHz pulse repetition frequency high-power thin-disk oscillators. In strong contrast with most typical ultrafast high-power lasers based on master-oscillator power amplifier schemes that require multiple amplification stages to reach the desired high-average power levels, modelocked thin-disk oscillators are single box units directly producing ultrashort pulses at hundreds of watts of average power and operating at multi MHz pulse repetition frequency, offering a number of potential advantages. We will summarize here their operation principle, the latest state-of-the-art in the current context of high-power ultrafast lasers, as well as discuss future directions and challenges. Our goal with this short review is to bring this promising technology to the attention of the material processing community and highlight its unexplored potential.

We note also here that a recent field of research is currently emerging, that makes use of much higher pulse repetition frequency lasers in the GHz regime for material processing in the ablation cooling regime [12]. Whereas this is an extremely interesting direction, it is out of the scope of this manuscript, where we focus on traditional material processing at high repetition rates. It is however worth noting that this field could also become relevant for the technology that we are discussing here in the near future.

### 1.2 Thin-disk laser basics and state-of-the-art of continuous wave and amplifier setups

**Figure 2:**
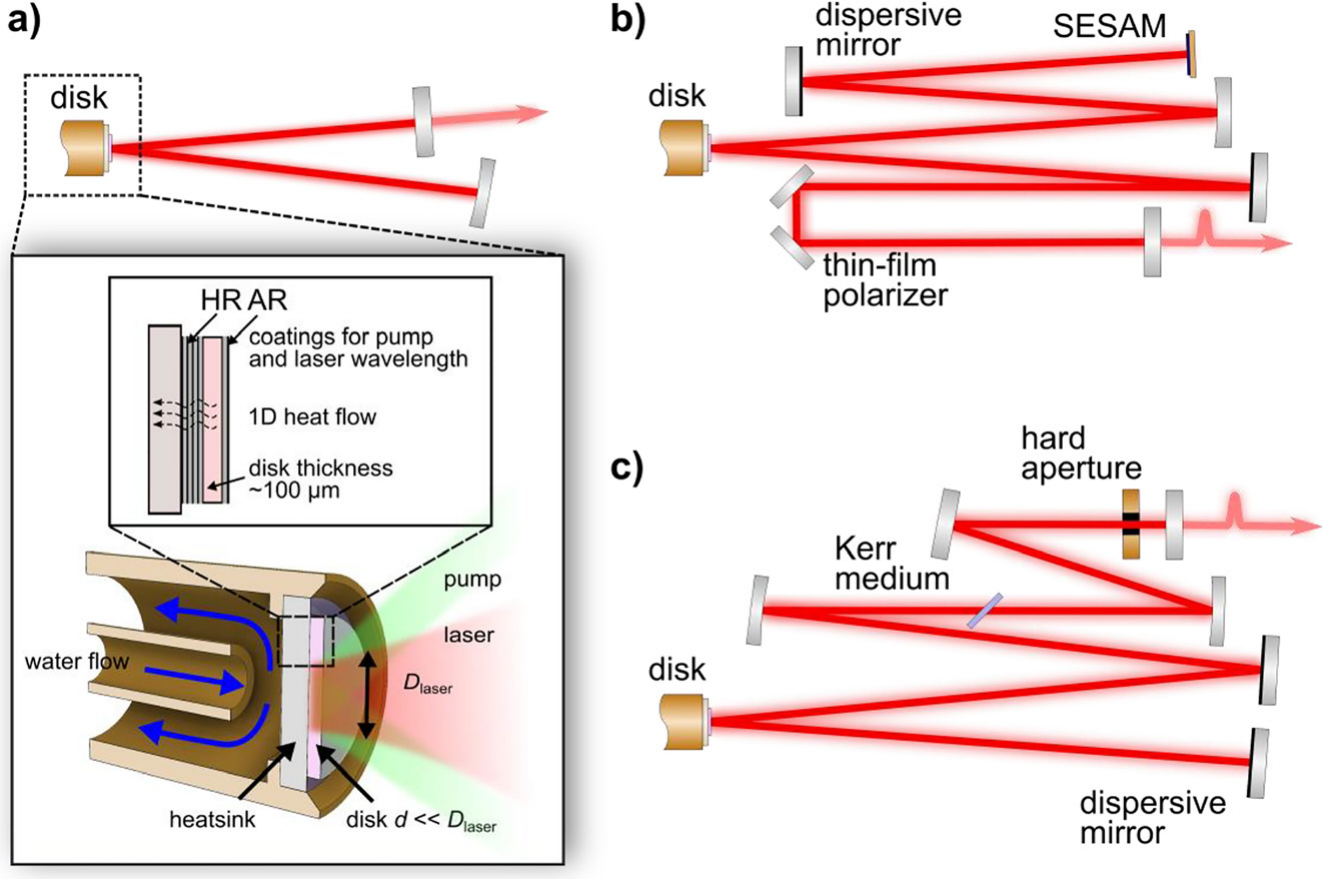
Thin-disk laser concept and schematic illustration of modelocked TDLs, a) simple schematic of a *cw* resonator with disk as a folding mirror; zoom-in of the disk gain element and cooling concept, b) typical SESAM-soliton modelocked TDL layout showing two dispersive mirrors illustrated to compensate for intracavity SPM, c) typical Kerr-lens modelocked TDL layout with Kerr-medium separated from the gain and hard aperture.

The key element in a thin-disk laser (TDL) [13] is the disk-shaped gain medium, which can be used in a resonator or amplifier. In Figure 2a) we schematically illustrate such a thin-disk resonator layout and the main concept behind the thin gain element. In this scheme, the gain medium has a very small thickness with respect to its diameter, with a typical factor higher than 10 between these two dimensions. The back side of the disk is coated with a highly reflective dielectric mirror for both the pump and laser wavelengths and contacted on an appropriate heatsink (most commonly diamond). The front side is antireflection coated for the same wavelengths, and can therefore be used in reflection inside a resonator. In this way, if a sufficiently large pump area is applied, the gain medium can be efficiently water-cooled through the backside and the heat-flow is one-dimensional in the direction perpendicular to the disk. Because of the short length of the gain medium, a correspondingly small single-pass absorption and gain are typically achieved in this geometry: both efficient pumping of thin-disk shaped solid-state laser media and significant gain require multiple-passes through the gain medium. Commercially available industrial pumping modules with up to 72 pump-light passes allow us to efficiently pump the gain medium in this geometry despite the low single-pass absorption. In the same fashion, multiple passes can be used either in a resonator (oscillator or regenerative amplifier) or by geometric multiplexing or a combination of both to reach high gain per roundtrip and not being limited by the low gain per pass inherent to this geometry [14], [15], [16], [17], [18].

In this thin-disk geometry, scaling of the average output power can be achieved by increasing the pumped area and pump power by the same factor, and excellent beam quality can be achieved at high power levels. In fact, only very low residual thermal aberrations remain at very high pump intensities. The spherical part of the aberrations can be compensated with the resonator design in a straightforward way [19]. Alternatively, deformable mirrors can be used intracavity to compensate higher order aberrations [20, 21]. In continuous-wave (*cw*) operation, powers well beyond 15 kW have been reached from a single-disk oscillator by the company TRUMPF [22], nearly single-fundamental operation (*M* ^2^ = 1.76) was demonstrated at 10-kW average power using two polarization-combined oscillators and one multi-pass amplifier system [18] and a 4-kW fundamental mode oscillator with an *M* ^2^ of 1.4 was achieved [23]. All these results were achieved with Yb-doped garnets, emitting at 1030 nm wavelength, but there is continuous interest in the scientific community to explore other materials and dopant ions for extending these records to other wavelength regions as we will discuss in a following paragraph.

The impressive performance of TDLs in *cw* operation can be extended to ultrafast operation in a straightforward fashion. In fact, the disk gain geometry, with large spot areas on the gain element offers great advantages for amplification of ultrashort pulses with record high pulse energies, using either regenerative amplifiers or multipass amplifiers. Whereas fiber chirped pulse amplifiers reach at the moment the highest average power from any ultrafast laser system [5], pulse energies reachable with the thin-disk technology – in particular with a single gain unit without coherent combination – remained unsurpassed. Latest advances in this area have been recently reviewed [24], and recent record holding systems include for example the demonstration of 2-kW average power ps-long pulses at 20-kHz pulse repetition frequency [25] as well as energies in the multihundred mJ regime [3, 17, 26] using regenerative amplifiers. Thin-disk multipass amplifiers used as booster amplifiers have also seen spectacular progress reaching the kW level with large parameter flexibility [15]. The latest experiments led to the demonstration of ps pulses with 720-mJ pulse energy at 1-kHz pulse repetition frequency [27] and of up to 2.45 kW at a pulse repetition frequency of 300 kHz, corresponding to a pulse energy of more than 8 mJ, and a pulse duration below 8 ps [28]. Besides the fundamental wavelength at 1030 nm, these systems have also been frequency doubled and tripled to extend record power ultrafast performance into the visible region. For example in [29] a record high-power kW-class ultrafast green laser is demonstrated extending the possibilities for material processing. In fact, many materials exhibit lower ablation thresholds at shorter wavelengths as a result of typically higher linear and nonlinear absorption coefficients. Together with a better focusability, a larger variety of materials can be processed more efficiently for example metal [30, 31], glasses [32, 33], ceramics, thin films [34] and semiconductors [35].

The amplifier systems described above define nowadays the state-of-the-art in terms of average power and pulse energy with ultrashort pulses in the disk geometry. Most efforts so far have however been dedicated to realizing these high-average power levels in systems with very high pulse energy in the mJ to hundreds of mJ regime, i.e. at tens to hundreds of kHz pulse repetition frequency. For even higher pulse repetition frequency in the MHz region, directly modelocked thin-disk oscillators, which will be described below, are a compelling alternative to more widely applied fiber-based systems in this power/pulse repetition frequency class, possibly also in combination with multipass booster thin-disk amplifiers in the future, for reaching the >10 kW average power regime. In fact, as discussed above, nearly-fundamental mode *cw* kW oscillators can be achieved in the thin-disk geometry [18, 36]. Together with the small thickness and large areas on the disk, leading to low nonlinearities, this opens the door to kilowatt-class modelocked oscillators, which is in strong contrast to the fiber and slab geometries. We will discuss this promising technology in the next section.

## 2 Modelocked thin-disk oscillators

### 2.1 Concept

As discussed above, we focus here our attention on one specific technology among high-power ultrafast laser systems that is particularly promising for applications where high pulse repetition frequency in the MHz regime is desired. In the thin-disk geometry, a TEM00 high-power oscillator is fundamentally modelocked to generate ultrashort pulses emitted at a pulse repetition frequency defined by the length of one round-trip in the resonator. A schematic of typical *cw* and modelocked oscillator layouts is shown in Figure 2. For the most commonly used standing-wave resonators (where the gain disk is used as a folding mirror) the length of the cavity is half of the roundtrip length. Single-mode resonator lengths are typically in the order of a few meters for standard disks with few mm- aperture, resulting in a natural operation range in the 10s of MHz regime and the resonator length can be increased at a given average power using simple reflective telescopes or Herriott-type multipass cells to generate higher pulse energies [37, 38].

Obtaining robust fundamental-mode resonators for modelocked TDLs remain one of the most critical design challenges for these laser systems. In fact, stable modelocking requires robust single fundamental-mode operation and it remains unclear whether the latest kW *cw* systems fulfill this requirement with an *M* ^2^ > 1.4. Some of these challenges have been discussed in previous work: in [19] the difficulties in designing resonators with large mode areas with narrow stability zones for modelocking and in the presence of thermal lensing and in [39] the relevance of the gas-lens effect for kilowatt-class oscillators. Compensation techniques such as deformable mirrors based on pneumatic actuation has been implemented in *cw* oscillators which generate kW average output power with nearly diffraction-limited beam quality [20, 21]. Nevertheless, aspects such as resonant transverse-mode coupling [40] have not been thoroughly addressed in the case of disk lasers and much of the resonator designs are still designed with basic empirical guidelines such a simple pump/laser beam overlap optimization.

For modelocking of these high-power resonators, semiconductor saturable absorber mirror (SESAM) [41] or Kerr-lensing in a separate plate in the resonator [42] are most commonly used for starting and stabilizing modelocking, and pulse formation is dominated by soliton pulse formation [43] where self-phase modulation (SPM) and dispersion must balance each other at each roundtrip of the pulses throughout the resonator. SPM originates from all the nonlinear elements (Kerr medium, disk, air, etc.) seen by the pulses within one roundtrip in the resonator, and energy scaling is limited by the maximum tolerable level of nonlinearity before pulse breakup effects occur. A schematic representation of the typical resonator layout for both Kerr-lens modelocked (KLM) and SESAM-modelocked TDLs is presented in Figure 2(b) and (c) – showing that the principle of such an oscillator is no different than that of a low power modelocked oscillator. The complexity of such an oscillator in a real implementation, however, can be much higher while trying to power scaling the output power or pulse energy, for example when requiring vacuum chambers to reduce the nonlinearity of air [44, 45]. Attempts have been made to operate these lasers in chirped pulse regime to allow for higher pulse energy, but only with limited success [46, 47]. An interesting path that has been suggested in [48], is the use of other modelocking regimes commonly used in modelocked fiber lasers, that are more tolerant to strong nonlinear effects for pulse energy scaling. However, these approaches remain so far undemonstrated. The most successful approach so far to achieve high energy levels (>10 µJ) has been nonlinearity reduction within one resonator roundtrip in SESAM soliton modelocked or Kerr lens modelocked (KLM) TDLs, for example by operating the oscillator in vacuum to reduce the nonlinearity introduced by air [45, 49], and/or reducing the intracavity peak power to a minimum by using active multipass cell technique [16, 44], or by introducing an intracavity phase-mismatched second-harmonic-generation crystal which provides negative SPM [50].

### 2.2 State-of-the-art

**Figure 3:**
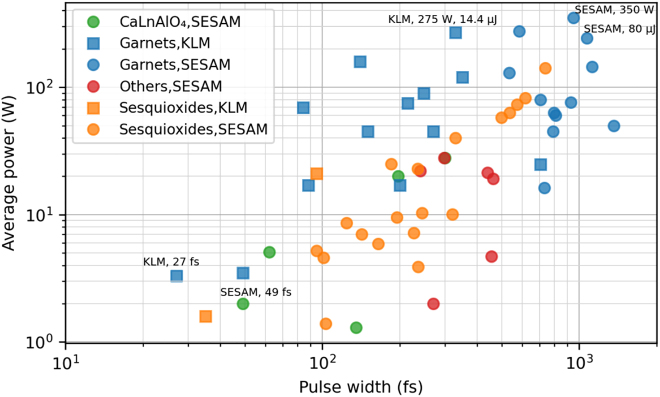
Overview of 1-µm MHz repetition frequency Yb-doped modelocked thin-disk oscillators.

The first modelocked TDL was demonstrated in the year 2000 at the ETH Zurich by the group of Prof. Keller, achieving 16.2 W from a 700-fs Yb:YAG modelocked oscillator [51]. This result set the ground for fast progress in the achievable levels in the following 20 years, consistently achieving orders of magnitude higher average power and pulse energy than any other ultrafast oscillator technology (see Figure 3) and reaching comparable levels to advanced high-power amplifiers operating at MHz pulse repetition frequency. Most of this progress has been achieved using Yb-doped garnets – following the trend of achievable levels in *cw* operation, therefore we focus here on the state of the art of TDLs based on Yb-doped materials and emitting around 1030 nm. Nevertheless, interest continues to find other suitable gain material for this geometry. We discuss some of these efforts in the next paragraph.

The latest state-of-the-art of the technology is shown in Figure 3 and some record-holding system parameters presented in Table 1. The highest average power reported so far is 350 W with 940-fs pulses and 39-µJ pulse energy [52], and the highest pulse energy 80 µJ in 1-ps long pulses at 3-MHz pulse repetition frequency and 242 W of average power [45] using SESAM soliton modelocking. Comparable average powers and peak powers were later on reached with Kerr-lens modelocking (KLM), where 270 W with 330-fs pulse duration have been reached [53] and later on 155 W with 140-fs pulses [54]. Naturally, Kerr-lens modelocked TDLs reach shorter pulse durations, which is mostly relevant for scientific applications, and significant efforts continue to be carried out in this direction by pushing the performance in this operation regime [55]. Focusing on the parameter range of interest for applications of material processing, pulse energies >10 µJ were achieved for the first time in 2008 at 3 MHz pulse repetition frequency using a helium-flooded resonator to reduce the intracavity nonlinearity of air [37], followed closely by the demonstration of >40 µJ, ps-pulses using the active multi-pass cell approach in air [14, 44] and the demonstration of 80-µJ ps-pulses at 242 W of average power in an evacuated environment. More recently, the same active multipass cell approach has been applied to KLM technique allowing reaching 13.2-µJ pulse energy with 290-fs long pulses in air [16].

**Table 1:** Representative results of Yb-doped modelocked thin-disk oscillators (bold indicates a record-holding system for the corresponding parameter for each of the two modelocking techniques).

Gain medium [Ref]	Modelocking method	Output power [W]	Pulse duration [fs]	Pulse repetition frequency [MHz]	Pulse energy	Wavelength [nm]
Yb:YAG [52]	SESAM	**350**	940	8.88	39 µJ	1030
Yb:YAG [45]	SESAM	242	1070	3.03	**80 µJ**	1030
Yb:CALGO [60]	SESAM	2	**49**	64.8	30 nJ	1050
Yb:YAG [53]	KLM	**270**	330	18.8	**14.4 µJ**	1030
Yb:YAG [55]	KLM	3.3	**27**	17.1	193 nJ	1030
Yb:CALGO [61]	KLM	0.15	**30**	124	1.2 nJ	1048

It is important to note that although this performance range can also be achieved with amplifier systems (see Figure 1), there are several potential advantages of the direct oscillator approach, however keeping in mind that most of these advantages have not yet been explored in detail in applications, since these systems are fairly novel, laboratory-based setups [56], [57], [58], [59]. Possibly the most relevant advantage is their amplifier-free operation providing a path for robust, compact and low-noise laser systems when developed into industrial-grade systems, which however remains to be demonstrated. In fact, most recently demonstrated thin-disk oscillators were achieved in laboratory settings and systems were not developed for long-term stability. Therefore amplitude noise is typically limited by technical noise (cavity vibrations, air turbulences, thermal drifts). These could easily be suppressed with active beam stabilization, controlling turbulences of the air with enclosed sealings, etc. With respect to stability of the laser system, it can be also expected that using geometries with higher gain per roundtrip at lower intracavity power [14, 44], these noise sources will be smaller. In the ultimate limit of a quantum noise limited system, high-*Q* resonators with low-loss should provide an advantage. We note however, that no thorough study so far has conclusively shown the potential of these lasers for low noise operation – this will most likely be demonstrated via experiments using sensitive detections schemes using the above-mentioned extra measures for noise reduction. For material processing, this will be less relevant and long-term stable operation will be more critical to address. Another advantage of thin-disk oscillators is that they provide extremely clean, sech^2^-shaped transform-limited pulses, with narrowest bandwidths for a given pulse duration and peak power. This generally simplifies and increases the efficiency of subsequent nonlinear setups such as nonlinear pulse compression or frequency conversion schemes, which become increasingly relevant for material processing.

## 3 Current research directions and challenges

### 3.1 Average power and pulse energy scaling

There are no fundamental road-blockers that prevent scaling of this technology to the kilowatt level and beyond. This should enable the demonstration of modelocked oscillators with tens to hundreds of microjoules of pulse energy at tens of MHz pulse repetition frequency. It is the opinion of the authors that this next step will most likely be achieved firstly using high-power SESAM modelocking technology [62], [63], [64], [65], and using Yb-garnets as gain materials, thus achieving pulse durations >500 fs because SESAM modelocking relaxes several constraints in resonator design, that should facilitate this next step to be realized in the near future. In fact, in a KLM laser, self amplitude modulation (SAM) and SPM are coupled to the resonator geometry, making it extremely sensitive to thermal variations in the resonator design. These often unknown thermal variations (i.e. thermal lensing) occur not only in the disk, but also in intracavity mirrors and other cavity elements, and become increasingly severe as the average power is increased, and the spot size on the resonator elements becomes power-dependent. Furthermore, at a given pulse peak power, KLM lasers operate at significantly higher intracavity nonlinearity, since this is required to achieve significant SAM, thus significantly higher amounts of dispersion per roundtrip is needed to achieve modelocking. Dispersive mirrors on the other hand are known to introduce additional thermal effects as compared to regular high-reflectors [38]. In the case of SESAM modelocking, these constraints are significantly relaxed, since SPM and SAM are decoupled from each other and from the resonator design, provided that surface quality and thermal effects are within tolerable values of the resonator. Reduction of the intracavity nonlinearity will most likely require operation in a medium-vacuum environment, also relaxing the detrimental gas-lens effect [39], possibly in combination with a multiple gain-pass geometry such as implemented in the latest power scaling step [52].

Another major challenge of high-power modelocked lasers using SESAMs is thermal effects due to very low residual absorption in the semiconductor absorber. State-of-the-art SESAMs designed for high-power lasers typically have rather low nonsaturable losses of ∼0.1% [62]. However, as the average power of thin-disk oscillators increase towards the kW level output power and intracavity levels increase accordingly to the multi kW level, thermal lensing becomes increasingly relevant and can have a strong influence on the resonator stability, particularly when using large-mode areas that are more sensitive to thermal gradients [63]. For example, with 10-kW intracavity power, 0.1% nonsaturable loss leads to 10 W of heat which can become significant if special attention is not paid to proper heatsinking. Mitigation strategies have included improved heat removal by removing the SESAM substrate [63], using thinner substrate SESAMs and different contacting and bonding techniques to the heatsink [52] or the use of a top sapphire substrate for thermal lens control [65]. This type of systems with hundreds of fs pulse duration, kilowatt average powers, and tens of microjoules of pulse energy will be ideally suited to be deployed in material processing applications at high repetition rates.

One potential weakness of this technology for this application is that the pulse repetition frequency of a modelocked thin-disk oscillator is directly related to the cavity length, thus is not as flexible as in regenerative amplifier where the pulse repetition frequency can be relatively freely adjusted via the Pockels cell switch that sets the number of gain roundtrips. Pulse repetition frequencies in the order of 3 MHz (corresponding to a cavity length of 50 m) have been achieved with long-cavity thin-disk oscillators with an intracavity Herriot-type multipass cell [45, 56], and in active multipass cells [44], but their more standard repetition rate is in the 10–100 MHz range as shown in Figure 1. In processing applications, these systems are thus suitable for high speed polygon scanner systems, which support high rate laser processing using up to 40-MHz repetition frequency lasers [11], rather than more conventional scanner systems like galvanometers. However, this reduced pulse repetition frequency flexibility can be partly circumvented by using these systems in the burst mode, with flexible burst durations. In fact, for few 10s of MHz and even up to GHz pulse repetition frequency laser systems, burst operation allows a combination of quasi-*cw* heating effects to support rapid etching rates and ultrafast laser interactions for clean ejection of material [66] or to reaching the so called ablation-cooled regime [12]. Compared with other amplifier-based systems with high repetition frequency such as fiber-laser systems where a low-power AOM is typically used before the main amplification stages to generate bursts at high average power [67, 68], thin-disk oscillators requires Pockels-cell which can support several 100 W average power and high repetition rates. Currently, the state-of-the-art in terms of power handling of high repetition rate BBO-crystal based Pockels cells is >500 W at approximately 250 kHz [69]. For much higher repetition rates of tens of MHz, RTP crystal can also be applied with the potential to sustain the 100 W average power level [69]. Further progress is expected in this area as interest in material processing at increasingly high repetition rates also grows.

### 3.2 Pulse duration: gain materials for 1 µm operation

While the soliton modelocking regime allows for a certain degree of freedom to adjust the pulse duration by changing the intracavity SPM or pulse energy, this is usually impractical and oscillators are designed for shortest possible pulses at highest power. Long pulse operation (i.e. several ps) is typically limited by spatial hole burning [70]. Most results discussed so far were achieved using industrial-grade Yb-doped garnets which are already mature in the disk geometry. Using these gain materials, pulse durations of several hundreds of femtoseconds are accessible at highest power and energy levels, see Figure 3. Although many material processing applications do not require significantly shorter pulses than this, a variety of studies shows that shorter pulses <100 fs [71] have unique advantages, making use of strong nonlinear absorption (i.e., 2, 3-photon absorption) of the materials [72]. We therefore summarize a few highlight results in the area of reaching shorter pulse durations with modelocked TDLs. Please note that since our goal is to describe the possibilities offered by the thin-disk technology, we limit the discussion to recent results in shortening the pulse duration directly achievable from the oscillator. However, important research efforts are also currently dedicated to efficient external pulse compression of this and other high average power ultrafast laser systems. For the sake of completeness, we mention here one of the most promising techniques, which is the use of efficient multipass cell compression stages [73], [74], [75], [76].

The main key parameter to ultimately reach pulse durations well into the sub-100 fs regime with comparable power levels than the state of the art (multihundred watts and beyond) and pulse energy levels of several tens of microjoules is a wide gain bandwidth, which can be reached using other host materials for the Yb-ion. Several potential Yb-doped broadband materials for TDLs have been reviewed in [77]. As shown in Figure 3, various hosts have been studied for this purpose such as sesquioxides Re_2_O_3_ (Re stands for Y, Lu or Sc) [78] or CaGdAlO_4_ (better known as CALGO) [79, 80]. These materials typically have a much broader gain bandwidth compared to garnets and have already demonstrated very short pulse generation with bulk oscillators [81]. However, stringent requirements on the material quality of large sized samples, and good anisotropic thermal performance required for the disk geometry has so far generally limited their power scaling in ultrafast thin-disk operation beyond few tens of watts [79, 82].

Only one material other than Yb:YAG has been so far successfully used to generate >100 W average power, that is the sesquioxide material Yb:Lu_2_O_3_ [83]. In comparison with garnets, this material has very high thermal conductivity (∼11 W/m·K [84]) with nearly double the emission bandwidth (∼12 nm), and is an isotropic material potentially suitable for ultrashort pulses and very high power operation simultaneously. Pulses as short as 35-fs have been demonstrated with an average output power of 1.6 and 10.7 W were achieved with 88-fs pulses [82]. However, due to the rather high growth temperature and complex crystal growth method [78], obtaining high quality large scale single crystals for TDLs remains a challenging task. Recent developments have demonstrated that sesquioxide ceramics are becoming a potential alternative to single crystals [85, 86]. Aside from this impressive recently reported first *cw* performance, in modelocked operation 3.7 W were demonstrated with pulse duration of 98 fs [86].

Using Yb:CALGO, 300-fs pulse duration at an average output power of 28 W and 197-fs pulse duration at an average output power of 20 W were demonstrated early on with SESAM modelocking [79]. Later on, pulse durations as short as 62 fs were demonstrated with an average output power of 5.1 W [80]. With KLM, pulses as short as 30 fs has been demonstrated which is shortest pulses directly from a thin-disk oscillator [61], however, the average output power was limited to a very low 150 mW [61] which can also be reached in the bulk geometry [81, 87]. Several difficulties have made power scaling of this material more difficult than initially expected: on the one hand this material is uniaxial therefore; absorption, emission as well as thermal properties are not anisotropic, which limits power scaling with good beam quality as required for modelocking. On the other hand, the growth of defect-free material with high doping concentration required for disk lasers is still not systematically achieved and requires more detailed investigation. Nevertheless, we expect that further developments with this and other materials will continue in the thin-disk geometry as these novel materials become of better quality and more widely accessible.

### 3.3 Longer wavelengths

So far, our discussion was mostly restricted to ultrafast laser systems and TDLs based on Yb-doped materials, emitting in the vicinity of 1030 nm, where most power and energy scaling has been achieved. However, several applications could benefit from longer driving wavelengths with comparable performance, and thus the development of such high-power ultrafast sources in the 2–3 µm region is a very active topic of investigation in the laser community. In the context of laser material processing, this wavelength range could be of potential interest for processing of soft materials, such as laser welding of polymers [88]. In addition, silicon wafer production chains could also benefit from such systems for subsurface modification, which could be achieved more efficiently with fewer damages on the surface thanks to the nonlinear absorption of ultrashort pulses above 1.95 µm in Si-crystal [89].

**Figure 4:**
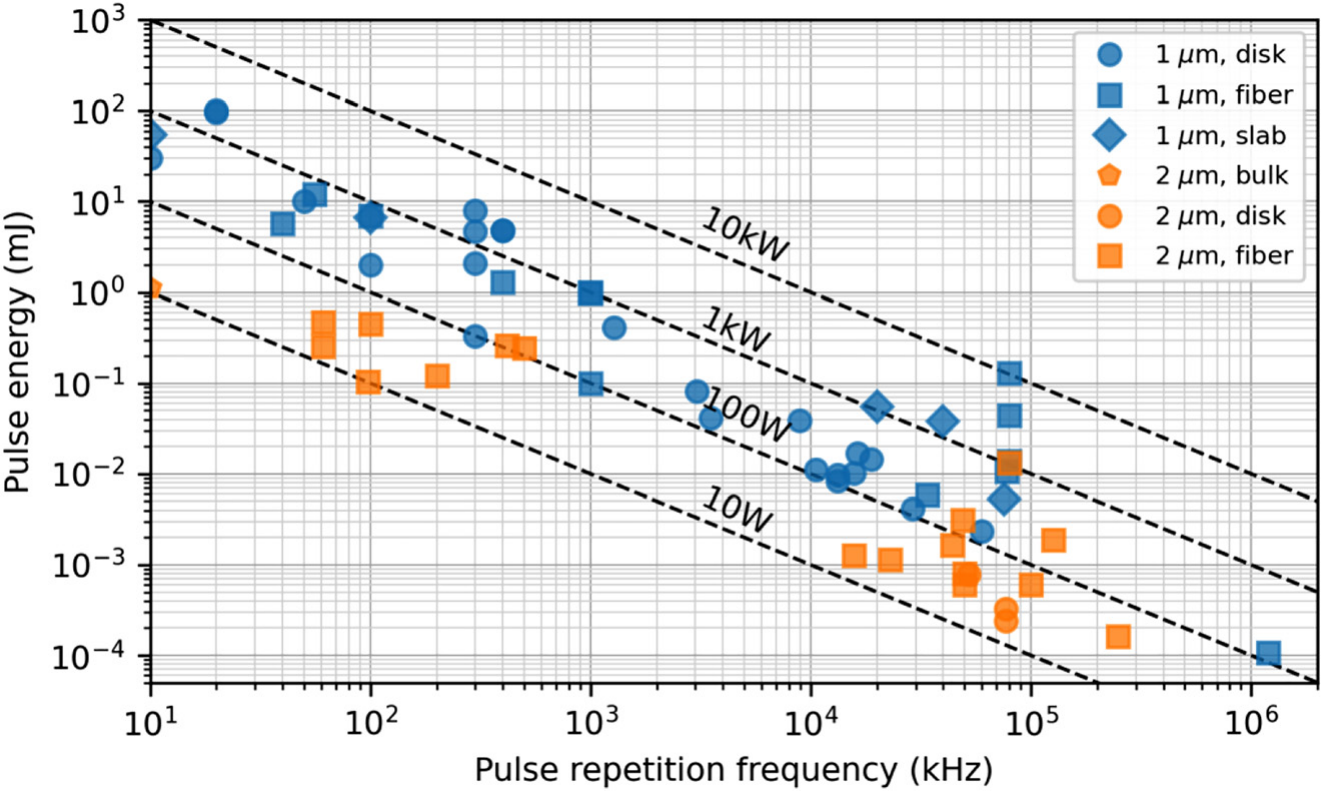
Comparison between 1- and 2-µm ultrafast lasers (including amplifiers and oscillators) in the bulk, fiber, slab and disk geometry.

Traditionally, this type of source was restricted to complex and inefficient parametric conversion stages, allowing reaching tens of watts of average power, using pumps with several hundreds of watts based on Yb systems described above [90]. A much more elegant and simple approach is to use gain media directly emitting in this wavelength range for high-power oscillators and amplifiers. As shown in Figure 4, many advances have been realized in bulk and fiber-based amplifier systems in this wavelength range. Some remarkable achievements in the wider area of high-power 2 µm ultrafast lasers are the demonstration of a Tm-fiber based chirped pulse amplifier systems, with an average power of 1060 W at 80-MHz pulse repetition frequency, corresponding to a pulse energy of 13.2 µJ [91]. At 500 kHz, 123 W of average power has been achieved as well [92]. Nevertheless, compared with ultrashort lasers in the 1-µm wavelength range, the average power of 2 µm ultrafast lasers are at least one order of magnitude lower as show in Figure 4, showing large potential for further progress. In particular, very few results have been achieved in this wavelength range with disk lasers – both oscillators and amplifiers – therefore representing an area of unexplored potential in ultrafast source development [93], which we aim to summarize here. Once again, we focus in the particular case of modelocking of high-power TDLs but many of the discussions presented below would also apply to disk amplifiers in this wavelength region.

The first pre-requirement for bringing the modelocked TDL state-of-the-art into this wavelength region is to find suitable gain media for power scaling in this geometry. In particular, materials with good thermal properties that remain high at high doping concentration, wide emission bandwidths, large emission and absorption cross-sections and pumping wavelengths compatible with high-power pumping are needed, and are challenging to find. Generally, finding such materials has gained significant attention in the last decade, but most research has so far focused on bulk laser modelocking with moderate power levels [94]. In the thin-disk geometry, most results in this wavelength region have only been achieved in *cw* operation and had not – until very recently – conclusively shown large potential. For direct emission in the vicinity of 2 µm, rare-earth Tm^3+^ (emission at ∼1.8–2.1 µm), Ho^3+^ (emission at ∼2.0–2.1 µm), and transition-metal Cr^2+^ (emission centered at ∼2.4 µm with bandwidth spanning ∼2–2.6 µm) doped-materials are potentially good candidates. We present here a summary of relevant *cw* and modelocking results achieved both with thin-disk and modelocked bulk lasers to illustrate the potential of further developments in this area. In Table 2, we summarize some of the relevant properties of gain materials which are promising for the thin-disk geometry in this wavelength region, following this discussion.

**Table 2:** Promising materials for SWIR ultrafast thin-disk oscillators and some relevant parameters.

Gain medium	Emission wavelength [µm]	Pump wavelength [µm]	Thermal conductivity [W/mK]	Thermo-optic coefficient (dn/dT) [10^−6^ K^−1^]	Shortest duration demonstrated (geometry)	Highest power demonstrated (geometry)^a^
Tm:YAG	∼2.01	0.79	8.8–13 [114]	∼7 (RT) [115]	3 ps (bulk) [116]	150 mW (bulk) [116]
Tm:Re_2_O_3_	∼2.1	0.79/1.61	11 [78]	∼9 [78]	41 fs (bulk) [117]	1 W (bulk) [118]
Tm:CALGO	<1.95	0.79	4.5–5.3 [119]	∼−8 (RT@1 µm) [120]	646 fs (bulk) [121]	330 mW (bulk) [122]
Ho:YAG	∼2.09	1.908	8.8–13 [114]	∼7 (RT) [115]	220 fs (thin-disk) [105]	40.5 W (thin-disk) [93]
Ho:Re_2_O_3_	>2.15	1.93	11 [78]	∼9 [78]	–	–
Ho:CALGO	∼2.02	1.93–1.95	4.5–5.3 [119]	∼−8 (RT@1 µm) [120]	52 fs (bulk Tm-co doped) [123]	376 mW (bulk Tm-co doped) [123]
Cr:ZnS(e)	∼2.4	1.6–1.7	27(18) [109]	54 (RT) [109]	<29 fs (bulk) [110]	2 W (bulk) [124]

^a^Modelocked oscillators.

Tm-doped materials can be pumped by high-power diodes at ∼790 nm, offering the possibility for high-power pumping, or alternatively, can be directly in-band pumped by Er-lasers at ∼1.6 µm. The first 2-µm *cw* TDL was based on Tm:YAG and was reported at the CLEO conference in 1998, only four years after the invention of TDL [13]. In this pioneering experiment, a 500-µm 10-at.% Tm:YAG crystal was diode pumped at 785 nm with eight passes and cooled by a Peltier-cooled heat sink achieving 2 W of *cw* power, and 18.3 W of peak power in quasi-*cw* regime [95]. Following this first result, several other Tm-doped materials were tried in the thin-disk geometry, such as Tm/Ho:KYW [96, 97], Tm:LLF [98], Tm:Lu_2_O_3_ [99] and Tm:KLuW [100]. In these early demonstrations, the highest power level achieved in *cw* operation was 21 W with Tm:LLF [98]. More recently, Tm:YAG was revisited and multimode *cw* operation up to 24 W (*M* ^2^ ∼ 11.7) was demonstrated. So far, no ultrafast operation of a Tm-doped disk laser has been demonstrated. In the bulk geometry however, several Tm-doped materials have been explored in modelocked operation: for example sesquioxides, CALGO and MgWO_4_, demonstrating pulse durations in the sub-100 fs duration with few tens of mW to few hundreds mW output power [94]. Among these materials, sesquioxides and CALGO based lasers are potentially good candidates for ultrafast TDLs. In 2018, the first sub-10 optical-cycle Tm-bulk laser has been demonstrated with Tm:LuScO ceramics [101]. This result illustrates not only the great potential of the Tm:Re_2_O_3_ sesquioxides for directly generating ultrashort pulses in 2-µm region, but also indicates possible modelocked operation with excellent optical quality large-size Re_2_O_3_ ceramics, which is particularly relevant for TDLs. One of the main difficulties with Tm-doped gain media in the thin-disk geometry is the rather limited absorption and gain cross-sections obtained in most host materials at reasonable doping levels, which limits the already small absorption and gain per pass in a sufficiently thin gain element. Higher doping concentrations are typically undesired due to a complex laser energy level scheme, prone to different detrimental energy transfer mechanisms.

**Figure 5:**
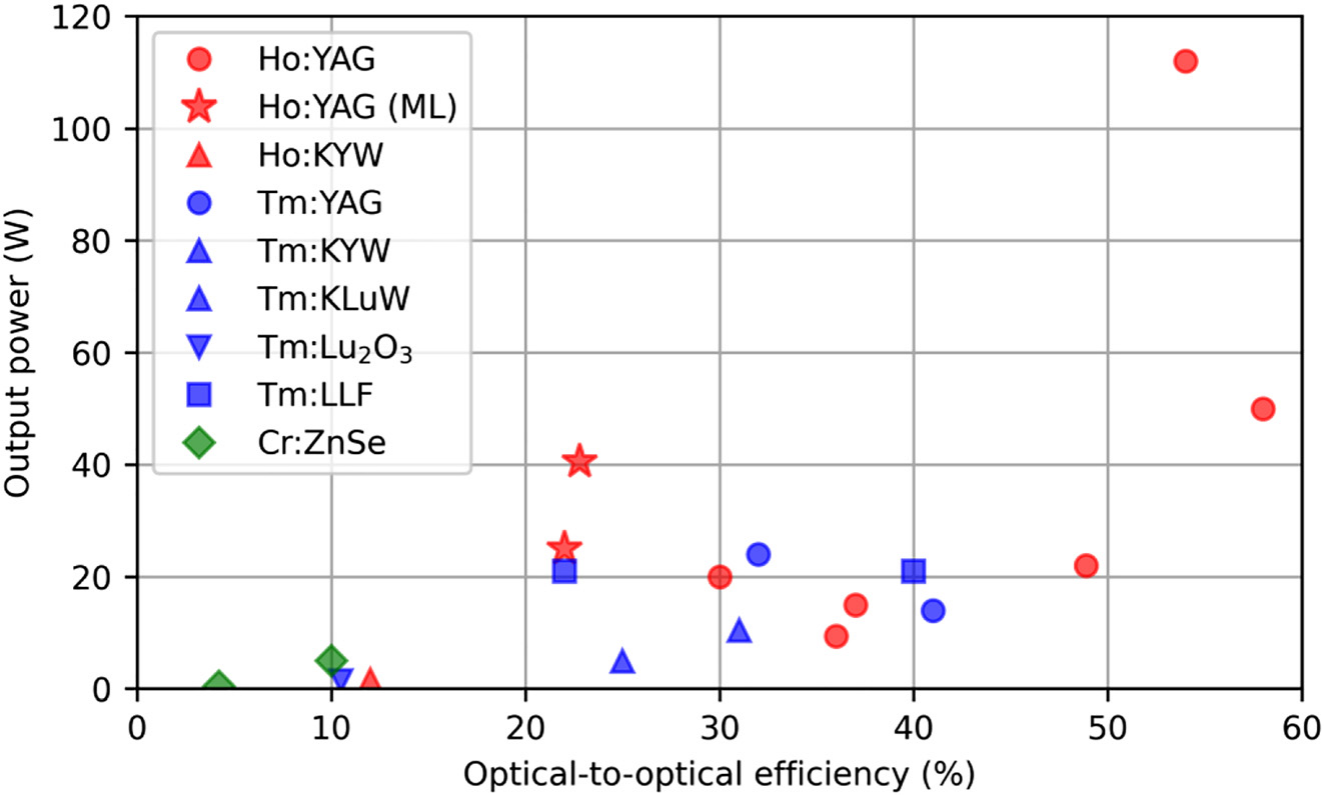
State-of-the-art of 2-µm cw and modelocked (with red star symbol) thin-disk lasers.

Ho-doped gain materials on the other hand have higher gain cross sections compared with Tm-doped materials, and exhibit a simpler three-level energy scheme with very small quantum defect, similar to Yb when in-band pumped at 1.9 µm, making it more promising for the thin-disk geometry. This pumping wavelength range can be accessed with high-power levels of hundreds of watts using commercial Tm-fiber lasers; however, power levels and price remain a bottleneck for multi kW operation when compared to high-power diodes. Ho-doped materials emit at wavelength above 2 µm (typically around 2.1 µm) which avoids many difficulties related to water vapor absorption at shorter wavelength. In 2006, the first Ho-doped TDL was demonstrated with 400 and 500-µm thick disks, ∼2-at.% Ho:YAG, reaching average output power of 9.4 W with an optical-to-optical efficiency of 36% [102]. A few years later, this result was extended to 22 W with InP diode-pumping [103]. In 2018, this material was revisited in the thin-disk geometry reaching 50 W in *cw* operation (*M* ^2^ ∼ 5.3) [104] and first ultrafast operation was achieved using KLM with an average power up to 25 W and 220-fs pulses [105]. More recently, our group demonstrated *cw* operation at 112 W with an *M* ^2^ ∼ 1.1 [106], and a record high average power of ∼40 W at a pulse duration of ∼1.66 ps [93] using SESAM modelocking. This latest progress shows that currently, Ho-doped materials appear to be the most promising for high-power ultrafast TDL operation, see Figure 5. Too much Tm ions with respect to other potential Ho-doped materials for the thin-disk geometry, most ultrafast bulk oscillators were accomplished by codoping with Tm ions [94], which can then be diode-pumped via the Tm ions and broader bandwidths can be exploited as their single ion counterparts, reaching pulse durations as short as sub-50 fs with an average output power of 121 mW [107]. However, Tm, Ho codoping method adds an additional degree of freedom which is the ratio between the Tm and Ho ions, which strongly affects lasing operation and depends on the host material, and therefore needs more detailed investigation before being attempted in the more complex thin-disk geometry.

Another very promising family of materials are the transition metals doped chalcogenides Cr:ZnS(e), which is known as the ‘MIR-Titanium:sapphire’ due to its ultrawide gain bandwidth [108, 109]. Modelocked oscillators based on Cr:ZnS(e) have so far exclusively been demonstrated in bulk laser form, but with very promising parameters. Pulse durations as short as three optical cycles (<29 fs) has been reached with 0.44-W average power at 100 MHz with KLM, corresponding to a spectrum as broad as 240 nm at −3 dB centered at 2.4 µm [110]. More recently, Watt-level sub-100-fs SESAM modelocking has been also demonstrated utilizing novel 2.4 µm GaSb-based SESAMs [111]. The main challenge for power scaling of the Cr:ZnS(e) based ultrafast laser systems in the thin-disk geometry is the rather strong thermal lensing effect caused by the large thermal-optic coefficient [109], compared with more standard Tm- or Ho-doped gain materials such as YAG. This difficulty is illustrated by early *cw* results with very moderate powers in the thin-disk geometry using Cr:ZnSe [103, 112, 113], reaching only 5-W output power.

In addition to the shared common challenges with the 1-µm region already mentioned in Sections 3.1, 3.2 and the less well-established gain material challenges discussed above, there are several other difficulties for extending the record performance of high-power TDLs to longer wavelengths: establishing high damage threshold optical components, saturable absorbers as well as availability of high-power pump sources. However, large interest in this area has resulted in some key developments that we believe will enable fast progress in the coming years. Recently, GaSb-based 2-µm SESAMs have been developed [125], and already resulted in several advances including our latest average power record using Ho:YAG [93]. High performance dispersive mirrors with large GDD and good thermal properties are also being developed for applications in modelocking, but also for general ultrafast dispersion control in this wavelength region.

## 4 Conclusion and outlook

To conclude, we have briefly reviewed the development of high-power, MHz pulse repetition frequency modelocked TDLs as promising candidate for use in high-rate material processing applications and reviewed current research directions for these sources that could become relevant for this application. In the 1-µm wavelength range, ultrafast thin-disk oscillators deliver up to 350-W average output power at MHz pulse repetition frequency, with pulse energies of tens of microjoules from one-box oscillators, that are already well-suited for processing for example of metallic materials with fast-scanning techniques, and the kilowatt level and beyond will be reached in the near future enabling even faster rates. The potential of this technology as a simple, one-box ultrafast oscillator for this application is promising and remains to be demonstrated. In particular, we expect industrial interest in this technology to grow, as very fast scanning methods continue to progress into ever increasing scanning speeds. Furthermore, this technology is an interesting seed source for existing multipass amplifiers, which could easily bring them to the >10 kW average power region at MHz repetition rates, enabling even more possibilities in this area.

As a future direction for these modelocked high-power TDL sources, we emphasize one area that we expect will develop fast in the coming years, that is the development of ultrafast TDLs emitting directly in the 2-µm wavelength range, which could expand the possibilities for material processing to other types of materials such as polymers, as well as fuel other potential areas of research.
